# Listening in: Orthopaedic Oncology Physicians' Perspectives on Implementation of Audio Recording/Artificial Intelligence Assist in Office Visits

**DOI:** 10.1002/jso.70237

**Published:** 2026-03-21

**Authors:** Megan Hsu, Annemarie K. Leonard, Sean McGarry, Tessa Balach

**Affiliations:** ^1^ Department of Orthopaedic Surgery and Rehabilitation Medicine University of Chicago Chicago Illinois USA; ^2^ Department of Orthopaedics University of Iowa Iowa City Iowa USA; ^3^ Department of Orthopaedics University of Nebraska Medical Center Omaha Nebraska USA

**Keywords:** artificial intelligence, audio recording, orthopaedic oncology, orthopaedics, surgical clinical visits

## Abstract

**Introduction:**

With rapid expansion of artificial intelligence (AI) in clinical documentation, responsible implementation of this tool is imperative in preserving the patient‐physician relationship. Orthopaedic oncologists were surveyed to assess their utilization of, and attitudes towards, ambient listening software.

**Methods:**

An anonymous, voluntary, IRB‐exempt survey (Appendix) was reviewed and distributed to members of the MSTS via the society's email listserv and distributed in paper and electronic formats to attendees at the AAOS Oncology Subspeciality day from March 14 to May 22, 2025.

**Results:**

Sixty‐three orthopaedic oncologists responded to the survey. Most (93%) practiced in an academic setting. Twenty‐seven percent reported using AI with a majority using Dax Copilot. Half of AI users noted a positive impact on clinical encounters, and one respondent reported a negative impact. Most AI users (86%) reported improved efficiency and accuracy in documentation and 40% reported saving 1–2 h per clinic day. Of non‐users, 71% were considering implementation.

**Conclusion:**

Although most orthopaedic oncologists are not using AI, the majority are considering implementation. AI users reported improvements in their documentation efficiency and accuracy. Further research is needed to understand the risks and benefits of this clinical tool from both providers' and patients' perspectives to guide responsible, widespread implementation.

## 1 Introduction

Documenting clinical encounters in an accurate and precise manner is paramount in delivering high quality patient care. Thus, all tools to aid physicians in documentation must be measured by their ability to do so while maintaining the patient confidentiality central to the physician‐patient relationship. It is well demonstrated that physician documentation burden contributes to overall physician burnout [1, 2, 3, 4, 5, 6, 7]. Some mitigate this burden either by use of dictation software or scribes [1, 4, 6]. Dictation software, while having decreased documentation time, still requires time of the provider outside of the immediate clinical encounter which can increase the risk of errors [6]. For example, errors result from recollection of the encounter when dictation is delayed to the end of clinic. In‐person scribes have the benefit of real‐time documentation however this technique requires another individual to be present thus incurring an additional cost, and accuracy is dependent on scribe training level and physician review.

There is a high degree of interest in employing ambient listening software in clinical documentation as it has the potential to be both a real‐time documentation tool while also not requiring an additional individual to be introduced in a physician‐patient encounter. However, with any new technology, the risks and benefits should be thoughtfully considered prior to widespread implementation. A recent systematic review demonstrated increased efficiency and decreased documentation times. Other studies reported an increased post‐encounter editing burden [7]. To the author's knowledge, there has not been a study assessing orthopaedic oncologists' usage of, and perspectives towards, incorporation of AI dictation into clinic visits.

## 2 Materials and Methods

An anonymous, voluntary, IRB‐exempt survey (Appendix) was reviewed and approved for distribution by the Musculoskeletal Tumor Society (MSTS) Membership Committee. It was distributed to MSTS members via email and American Academy of Orthopaedic Surgery (AAOS) Oncology Subspecialty Day attendees in paper and electronic formats from March 14 to May 22, 2025. No incentives were offered for participation in the survey. Anonymity was ensured by not collecting personally identifiable information such as participant names, email addresses, or IP addresses. Data collected was reviewed in aggregate, and the research team did not have knowledge of who responded to or did not respond to the survey.

## 3 Results

Sixty‐three orthopaedic oncologists responded to the survey. The response rate is 63 respondents of 394 surveyed equaling 16%. One respondent (#20) completed the demographic section and indicated they use AI, but did not complete the remainder of the survey. Their responses were included for the portions they completed. A majority (94%) practiced in an academic setting in comparison to a private practice setting. A majority of respondents (96.8%) practiced in the United States with only two respondents practicing outside the United States.

**Figure 1:**
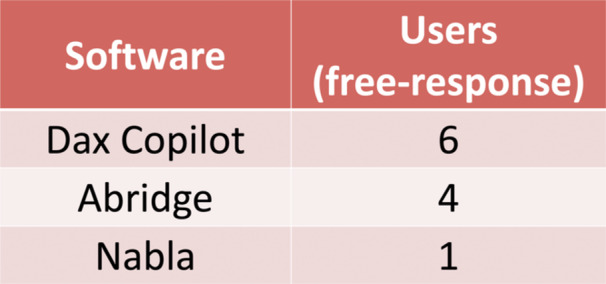
Chart of self‐reported software use.

**Figure 2:**
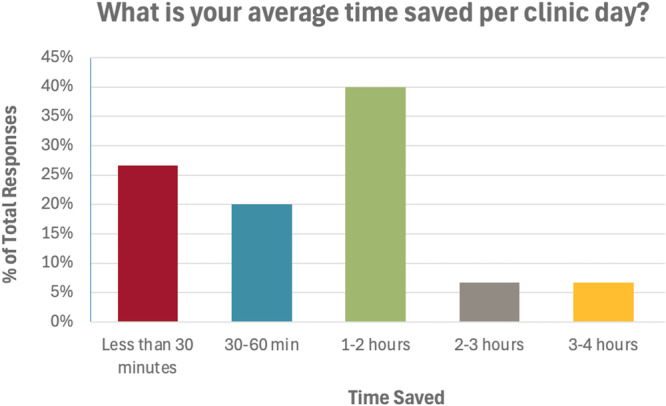
Time saved per clinic day.

Twenty‐seven percent reported using AI ambient listening software with a majority using Dax Copilot followed by Abridge (Figure 1). Of these, 38% utilized AI for every visit. Half of AI users noted a positive impact on clinical encounters, and one respondent reported an undisclosed negative impact. One AI user reported that patients rarely requested the software be turned off during an encounter. Most AI users (86%) reported improved efficiency and accuracy in documentation with 40% reporting saving 1–2 h per clinic day (Figure 2).

**Figure 3:**
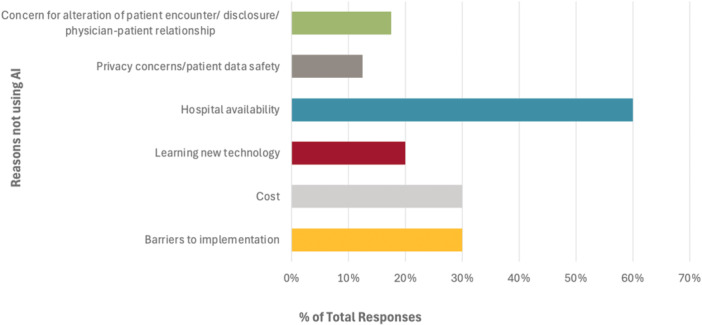
Barriers to AI implementation.

Of non‐users, 71% were considering implementation. Key barriers to implementation included hospital availability (60%), cost (30%), learning new technology (20%), concern for alteration of the patient physician relationship (18%), and patient data safety (13%) (Figure 3).

Free text comments included additional insights into potential barriers to, or concerns regarding, implementation. Five respondents expressed concern in the free text responses that AI ambient listening software may interfere with resident education as residents are currently writing and dictating the notes. Additional free‐text responses mentioned satisfaction with current work flow (3), needing more assessment of the tool (2), not considered beneficial (2), medicolegal concerns (1), billing downgrades (1), not adequately capturing nuances of thought (1), recent retirement (1).

## 4 Discussion

Documentation in the electronic medical record has been shown to contribute to physician burnout, and traditional dictation tools and scribes are useful but imperfect adjuncts. Scribe efficacy and accuracy depends on training and increases the number of people in the room for the patient encounter. AI ambient listening software is nascent to the orthopaedic clinic, but has the potential to increase accuracy of clinical documentation, decrease the number of people in the room for a clinical visit, and improve efficiency to potentially decrease physician burnout [8, 9, 10, 11, 12, 13]. AI users in our study reported improvements in their documentation efficiency and accuracy. Although most orthopaedic oncologists are not yet using AI, this study finds that the majority are considering implementation. Potential concerns include the accuracy of the AI software, hospital availability for implementation, cost, unknowns about how the patient‐physician relationship will be altered, and patient data safety.

Limitations of this study include those inherent to a voluntary survey study. The response rate is low at 16%. The low response rate may be due to survey fatigue or perceived low relevance to the recipient's practice. The data was self‐reported, which may lead to recall bias. Additionally, the study design, while attempting to reach potential respondents via email as well as in person with paper formats, is subject to non‐response bias. It is possible that the portion of musculoskeletal oncologists who did not respond to the survey exhibit different characteristics, qualities, or preferences than those that responded. Those individuals who did not respond may be unfamiliar with this tool, may not find the tool useful, or may not have been present at the AAOS Oncology Subspecialty Day. Respondents using AI may also have been more likely to respond to the survey, leading to a possible response bias or social desirability bias. There were only 2 international respondents which may be improved in future studies by sending the survey to international musculoskeletal oncology groups such as the International Society of Limb Salvage (ISOLS). Given the majority of respondents were US‐based academic respondents, results may not be generalizable to those practicing musculoskeletal oncology in private practice or internationally, although this was not the target audience for this study. There was one incomplete survey. We have fewer responses from those using AI regarding their experience. This may be due to less participants using AI overall, but this is uncertain. Finally, this survey was not previously validated or adapted from prior studies.

One respondent noted that a patient requested the ambient listening device to be turned off and a separate respondent described an unspecified negative impact on their patient encounters. Thus, we also need further information about how AI may alter the patient‐physician relationship specifically in the field of orthopaedics from both the physician and patient perspectives, including potentially improving the relationship by allowing more eye contact and natural conversation or, unintended negative consequences such as patients potentially withholding sensitive information during their visit due to privacy concerns with ambient listening software. In a small survey of 103 patients at a large academic center, patients reported possibly altering their conversations with their physician when discussing sensitive topics including mental health, sexual health, or illicit activities [14].

Thirteen percent of our respondents reported concerns regarding patient data safety. A quality improvement study demonstrated patients share this concern of potential mishandling of sensitive information despite being overall supportive of technology adaptation (14). Additionally, one respondent in our study reported medicolegal risks as a barrier to implementation of which there are many. For instance, it has yet to be adjudicated how discrepancies between an AI audio recording versus the documented medical record would be utilized by the judicial system, especially as physicians previously have been advised against keeping multiple forms of official documentation [15].

Five respondents cited concerns regarding impacts on resident education. This commentary highlights the critical balance of teaching trainees orthopaedic fundamentals in an environment of rapid technologic innovation. One perspective advocates for adoption of AI technology later in training to minimize the risk that early adoption has on trainees thinking critically and learning key foundational skills for their careers. Early overreliance on technology could impair a budding surgeon's ability to determine what is relevant for workup, diagnosis, formulation of an assessment and plan, and documentation [16]. Additionally, trainees may not learn to effectively navigate the EMR independent of AI for key information or may miss patient details that did not make it into EMR's AI rapid patient chart summarization tool. To put it simply, patients have expressed a clear desire for physicians to maintain oversight of this technology [16], but without developing skills independently of AI such as chart review, distilling a history and physical exam into pertinent positives and negatives, and using critical reasoning to make a differential diagnosis followed by a clear and concise assessment and plan, trainees may lose the ability to assess for and correct errors such as note bloat or diagnostic errors.

The counterargument is that residents are already using AI, and any attempt to curtail this would be impractical. The American Medical Association (AMA) has emphasized AI's ability to enhance human intelligence rather than replace it [17]. If one shields trainees from innovation, such as AI dictation, trainees may miss valuable opportunities to learn to critically appraise the tool's output and incorporate the tool responsibly. Using AI dictation may decrease note writing time in the clinic, allowing the learner to have additional time to ask questions of the attending and learn. Resident notes would more reliably be done at the end of clinic, allowing attendings to provide their attestation and close the visit more efficiently. It is paramount to train the next generation of orthopaedic surgeons to thoughtfully implement novel tools throughout one's career. AI, specifically regarding dictation software, holds a unique position with the ability to impact all facets orthopaedics regardless of eventual practice setting; thus, shielding residents from its implementation could also prove detrimental to their overall education. Future studies could also examine perspectives on whether AI ambient listening software improves or hinders resident education with regard to clinic note writing from the perspectives of both faculty and residents. Early systematic reviews on the topic of patient perceptions of physician utilization of these AI tools show patients overall accept the utilization of these tools, however, they want strong physician oversight in its use and implementation. Educating the next generation of physicians on AI utilization is important for responsible implementation of this tool [16].

## 5 Conclusion

This survey provides insight into MSTS members' current usage of, and attitudes toward AI ambient listening software. About one‐third of survey respondents are using AI, and about 71% of non‐users are interested in trying the technology. These results also demonstrate a potential decreased burden of electronic medical record documentation with 40% of AI users saving 1–2 h per clinic day. Responders' free‐text answers raised additional questions including the effect of the technology on the patient‐physician relationship and patient data safety. Further research is needed to understand the risks and benefits of this clinical tool from both providers' and patients' perspectives to guide responsible, widespread implementation.

## Funding

The authors received no specific funding for this work.

## Conflicts of Interest

The authors each list the following disclosures: Megan Hsu: no disclosures. Annemarie K. Leonard: Accreditation Council of Graduate Medical Education orthopaedic review board member, Ruth Jackson Orthopaedic Society Education board member. Sean McGarry: medical board of trustees for MTF biologics, panel member for National Comprehensive Cancer Network for bone sarcoma and soft tissue sarcoma. Tessa Balach: no disclosures.

## Synopsis

In this study, we surveyed the perspectives of orthopaedic surgical oncologists on the use of audio recording technology in clinical visits given the rapid expansion of artificial intelligence (AI) in clinical documentation and the importance of responsible implementation of this clinical tool. On average for those utilizing AI, orthopaedic oncologists found AI was a beneficial tool with minimal detrimental impacts on patient care which is a similar finding to recent studies of other physicians. Most orthopaedic oncologists who were not utilizing AI were considering implementing it in their practice.

## Figures and Tables

**Figure A1:**
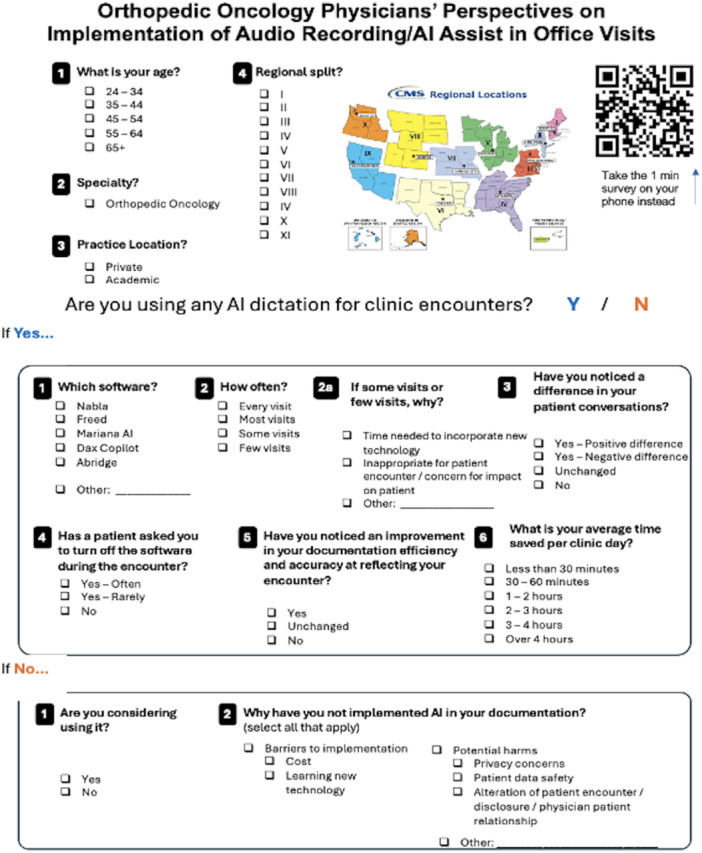
One‐page survey question in color.

## Data Availability

The data that support the findings of this study are available from the corresponding author upon reasonable request.

## 1

See Figure A1.
